# Low-magnesium, *trans*-cleavage activity by type III, tertiary stabilized hammerhead ribozymes with stem 1 discontinuities

**DOI:** 10.1186/1471-2091-6-14

**Published:** 2005-08-12

**Authors:** Donald H Burke, S Travis Greathouse

**Affiliations:** 1 Department of Chemistry, Indiana University, Bloomington, IN 47405-7102 U.S.A; 2 Department of Molecular Microbiology and Immunology, 471h Life Sciences Center, University of Missouri-Columbia, School of Medicine, 1201 Rollins Dr., Columbia, MO 65212-7310 U.S.A

## Abstract

**Background:**

Low concentrations of free magnesium in the intracellular environment can present critical limitations for hammerhead ribozymes, especially for those that are designed for intermolecular (*trans*) cleavage of a host or pathogen RNA. Tertiary stabilizing motifs (TSM's) from natural and artificial ribozymes with a "type I" topology have been exploited to stabilize *trans*-cleaving hammerheads. Ribozymes with "type II" or "type III" topologies might seem incompatible with conversion to *trans*-cleavage designs, because opening the loop at the end of stem 1 or stem 2 to accommodate substrate binding is expected to disrupt the TSM and eliminate tertiary stabilization.

**Results:**

Stem 1, together with single-stranded segments capping or internal to this stem, contains both the substrate-binding and tertiary stabilization functions. This stem was made discontinuous within the sTRSV hammerhead ribozyme, thereby separating the two functions into discrete structural segments. The resulting ribozyme, designated "RzC," cleaved its 13 nucleotide target substrate at MgCl_2 _concentrations as low as 0.2 mM at 25°C and 0.5 mM at 37°C. Under multiple-turnover conditions, nearly thirty turnovers were observed at the highest substrate:RzC ribozyme ratios. Similar stabilization was observed for several derivatives of RzC. Catalytic activity was diminished or eliminated at sub-millimolar MgCl_2 _concentrations for ribozymes with weakened or deleted tertiary interactions. Eadie-Hofstee analysis revealed that the stabilized and non-stabilized ribozymes bind their substrates with equivalent affinities, suggesting that differences in observed activity are not the result of diminished binding. Some of the stabilized and non-stabilized ribozymes appear to fold into a heterogeneous collection of conformers, only a subset of which are catalytically active.

**Conclusion:**

Hammerhead ribozymes with the "type III" topology can be converted to a tertiary, *trans*-cleavage design. Separating the stabilization and substrate recognition functions of stem 1 increases cleavage activity at physiological concentrations of divalent magnesium while retaining recognition of exogenous targets. *Trans*-cleaving ribozymes that exploit the tertiary stabilizing motifs of all natural hammerhead topologies can therefore be used in intracellular applications.

## Background

Self-cleaving hammerhead ribozymes contain three base-paired stems joined by a highly conserved core. Tertiary stabilizing motifs (TSM) of diverse morphologies between single-stranded elements at the ends of, or within, stems 1 and 2 increase cleavage activity at physiological concentrations of divalent magnesium ions *in vitro *and in cells [1-8]. This discovery has propelled a resurgence of interest in metal ion binding by hammerhead ribozymes [9,10] and in the use of intracellularly expressed ribozymes as gene-knockdown agents. Low magnesium concentrations in the intracellular environment can be a critical limitation for hammerhead ribozymes. Although the total intracellular concentration of divalent magnesium is approximately 3.5 to 8.5 mM, analysis of ^31^P chemical shift indicates that free Mg^2+ ^ranges from 0.2 to 1.2 mM and is generally between 0.4 to 0.8 mM depending on tissue type and physiological state [11-15]. Consistent with this view, the intracellular kinetic behavior of a hairpin ribozyme is more closely approximated by *in vitro *assays carried out at 2.0 mM MgCl_2 _than at 10 mM MgCl_2 _[16]. It is therefore important to define the ribozyme topologies and sequences that confer low magnesium activity.

**Figure 1:**
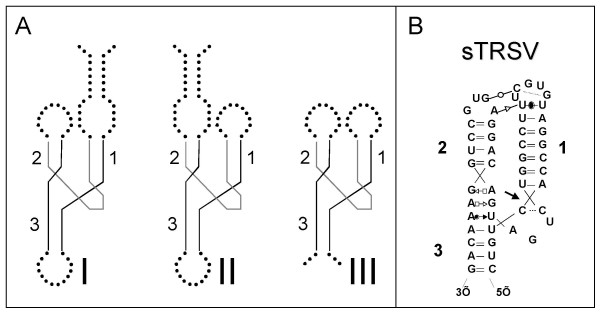
**A**. Types I, II and III hammerhead ribozymes. Peripheral regions shown as dotted lines contain the tertiary stabilizing motifs and can be of arbitrary sizes. Stems 1, 2 and 3 are indicated. **B**. The type III hammerhead ribozyme from sTRSV, showing tertiary interactions predicted from comparative sequence analysis, mutational data and computational modeling [2] (pairwise interactions depicted according to ref [22]).

Hammerheads are classified as being of type I, II or III according to whether the 5' and 3' termini reside within stem 1, 2 or 3, respectively (Figure 1A). The distinct connectivity patterns make these three types topologically non-equivalent. Tertiary-stabilized type I hammerheads, such as the SMαl ribozyme from *Schistosoma mansoni*, are readily adapted for *trans*-cleavage by opening the loop at the end of stem 3. In the SMαl ribozyme, however, nucleotides from both the substrate and ribozyme strands contribute to establishing stable tertiary interactions, significantly limiting the range of substrates that can be targeted for cleavage at physiological concentrations of Mg^2+^. We recently described hammerhead ribozyme RzB, which was derived from *in vitro *selections from a library of type I self-cleaving hammerheads. RzB carries an artificial TSM that is nearly independent of the sequence of the RNA fragment to be cleaved, freeing the experimental design from constraints encountered in ribozymes based on SMαl [4].

There have not been reports of using the type II or type III topology for low-magnesium *trans*-cleavage. Opening the loop at the end of stem 1 in these ribozymes to accommodate substrate binding is expected to disrupt the TSM and eliminate tertiary stabilization. We reasoned that type II and type III hammerhead ribozymes could nevertheless be used for *trans*-cleavage at physiological magnesium concentrations if the substrate binding function of stem 1 could be separated from the tertiary stabilizing function of the TSM carried within loop 1. To this end, we constructed *trans*-cleaving versions of the type III self-cleaving hammerhead ribozyme from the Tobacco Ring-Spot Virus satellite RNA (sTRSV) [17]. The functional separation was achieved by placing both the 5' end of the ribozyme and the 3' end of the cleavage substrate within stem 1. Ribozymes with a discontinuous stem 1 exhibited tertiary stabilization at physiological magnesium. We further demonstrate that this stabilization extends to cleavage of a human *ras *oncogene mRNA fragment.

## Results & Discussion

### Low-magnesium activity of *trans*-cleaving ribozyme with discontinuous stem 1

**Figure 2:**
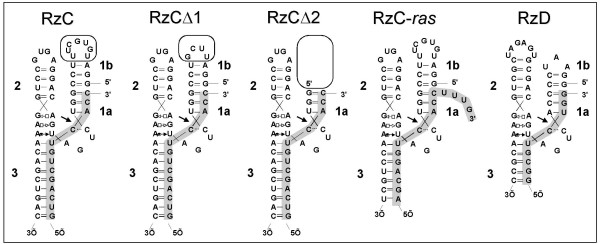
Ribozymes described in this study. Stems la, Ib, 2 and 3 are indicated. Substrate strands are shaded. Nucleotides involved in establishing the roles of tertiary stabilization in RzC, RzCAΔ1 and RzCΔ2 are boxed.

Ribozyme "RzC" was designed to preserve the endogenous tertiary interactions of the sTRSV hammerhead ribozyme. The distal half of stem 1 and all of loop 1 (terminal three base pairs and seven-nucleotide loop) are continuous sTRSV sequence. Substrate is recognized through base pairing with the proximal half of stem 1 (three base pairs) and all of stem 3. Stem and loop 2 are native sTRSV sequence, again to preserve tertiary interactions (Figure 2). Thus ribozyme RzC separates the two functions of stem 1 – substrate recognition and tertiary stabilization – into discrete structural domains.

**Figure 3:**
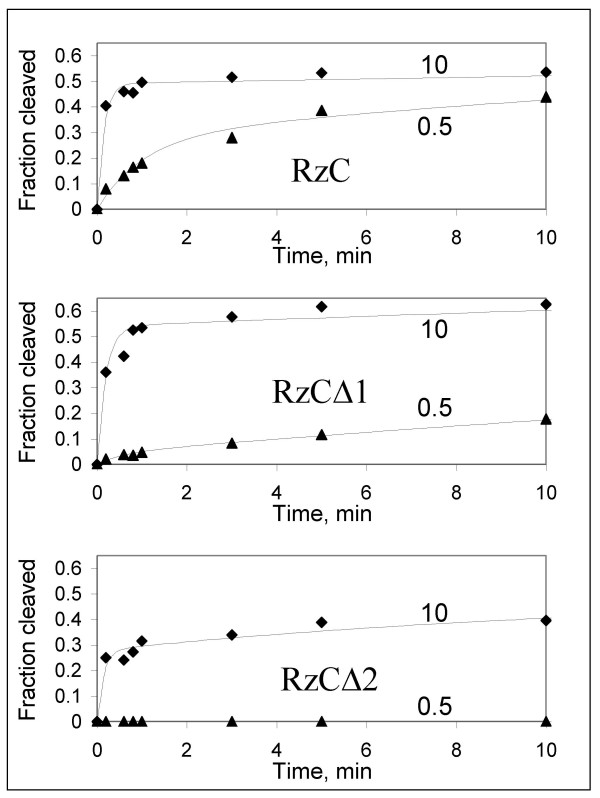
Magnesium dependence of substrate cleavage by discontinuous ribozymes with varying degrees of tertiary stabilization. Traces shown are for cleavage at 37°C in 10 mM (diamonds) or 0.5 mM (triangles) MgCl_2_. Data were fit to double exponential kinetic model as described in Methods, using best fit parameters given in Table 1.

The catalytic activity of RzC was measured in 10 mM MgCl_2_/pH 7.5/37°C for *trans*-cleavage of a 13-mer oligoribonucleotide denoted "substrate C." Greater than 40% of this substrate was cleaved within the initial 15 seconds, yielding an initial rate of at least 3.2 min^-1 ^(Figure 3, diamonds). Similar rapid cleavage was observed at 5 mM and at 2 mM MgCl_2 _(not shown). Importantly, RzC also cleaves substrate C at sub-millimolar Mg^2+ ^concentrations, yielding an initial rate of 0.3 min^-1 ^in 0.5 mM MgCl_2 _(Figure 3, triangles). When activity was measured at 25°C, ribozyme RzC continued to cleave rapidly at 0.2 mM MgCl_2 _(0.2 min^-1^) (Table 1). Much higher concentrations of MgCl_2 _are required to observe comparable rates for minimal ribozymes lacking TSM, suggesting that the TSM from sTRSV is functional in the context of a discontinuous stem 1.

**Table 1:** Kinetic parameters for discontinuous ribozymes derived from sTRSV

	[MgCl_2_], mM	f_∞_	f_a_	k_a_	k_b_	net init rate, min^-1^
RzC [25]	1.0	0.59	0.70	4.23	0.040	1.75
	0.5	0.54	0.58	2.03	0.070	0.65
	0.2	0.42	0.23	3.57	0.091	0.37
	0.1	0.11	0.62	0.92	0.010	0.06
RzC [37]	10	0.74	0.67	6.49	0.013	3.2
	5	0.63	0.77	2.31	0.022	1.1
	2	0.71	0.66	3.52	0.023	1.7
	0.5	0.73	0.38	0.98	0.040	0.3
RzCΔl [37]	10	0.68	0.78	3.92	0.12	2.09
	5	0.76	0.71	5.22	0.033	2.82
	2	0.76	0.57	1.51	0.062	0.67
	0.5	0.58	0.074	1.77	0.028	0.09
RzCΔ2 [37]	10	0.51	0.55	7.1	0.081	2.0
	5	0.66	0.41	3.1	0.025	0.85
	2	0.58	0.229	3.1	0.036	0.43
	0.5	n. d.	n. d.	n. d.	n. d.	n. d.

Kinetic parameters were determined for discontinuous ribozymes as described in Methods. Single-turnover reactions were performed at 25°C or 37°C (square brackets next to ribozyme designations), and the data were fit to a double exponential curve. n.d., no cleavage detected. No cleavage was observed for RzC at 0.075 and 0.05 mM MgCl_2 _(not shown).

Two variants of RzC were built to assess the contribution of the tertiary interaction modules to the observed magnesium dependence. Ribozymes RzCΔ1 and RzCΔ2 are identical to RzC outside of stem 1. In ribozyme RzCΔ1, the seven nucleotide terminal loop sequence 5' UGUGCUUU 3' is replaced with a stable tetraloop 5' UUCG 3', while in ribozyme RzCΔ2, the loop and terminal three base pairs are removed altogether. When reactions were monitored in 10 mM MgCl_2_, the deletions had little effect on initial rates, as all three ribozymes cleaved substrate C with initial rates of 2–3 min^-1^. Decreasing the MgCl_2 _concentration to 2.0 mM, however, produced a clear difference in initial cleavage rates, which were greatest for RzC and slowest for RzCΔ2. In 0.5 mM MgCl_2_, the initial rate of RzC was 3 times greater than that of RzCΔ1 (0.3 vs. 0.09 min^-1^), and no substrate cleavage at all was detected for RzCΔ2 (Figure 3 and Table 1). Interestingly, the rates observed for RzCΔ1 were closer to those of RzC than to those of RzCΔ2. It is possible that the intermediate activity seen for RzCΔ1 at low magnesium may be due to weak tertiary stabilization using alternative interactions between loop 2 and the stable tetraloop at the end of loop 1, although this possibility was not further explored.

The enhanced activity of RzC relative to RzCΔ1 and RzCΔ2 at submillimolar MgCl_2 _support the underlying hypothesis that the TSM stabilizes productive RNA folding. The fraction of the pre-annealed ribozyme-substrate complex that participates in the rapid phase of the cleavage reaction (f_a_) decreases only slightly for RzC at sub-millimolar MgCl_2_, while it drops precipitously for RzCΔ1 and RzCΔ2, suggesting that that fraction of the ribozyme that folds into a productive conformation is greater for RzC than for the other two ribozymes. The fraction of the ribozyme-substrate complex that can access the active conformation is given by f_∞_. (This estimation is actually a lower limit, as it assumes that the back reaction is negligible; a reasonable assumption given that the three-nucleotide 3' cleavage product is expected to dissociate rapidly, minimizing the possibility of re-ligation.) For reactions in which cleavage is observed, the value of f_∞ _is not strongly dependent upon MgCl_2 _concentration, although it is slightly lower for RzCΔ2 than for the other two. Together, these results indicate that the TSM sequence elements from the sTRSV hammerhead are providing similar tertiary stabilization in RzC.

### Substrate affinity unaffected by TSM deletions

To rule out the possibility that the rate differences among RzC, RzCΔ1 and RzCΔ2 might be due to differential substrate-binding affinity, multiple turnover cleavage kinetics were measured as a function of substrate concentration using 1 nM ribozyme and excess substrate (10 to 200 nM). These conditions yielded between 4 and 28 nM cleaved product, indicating between four and twenty-eight turnovers. The slope of an Eadie-Hofstee plot of the rate data gives the apparent affinity of the ribozyme-substrate interaction as the Michaelis-Menten constant, Km. When multiple-turnover reaction kinetics were monitored in 10 mM MgCl_2_, all three ribozymes gave comparable Km values (~40 nM for RzC vs. ~70 nM for RzCΔ1 and RzCΔ2). Thus, substrate affinity is dominated by base paring within stem 3, and is not substantially affected by either the tertiary docking interactions or by the stacking between stems la and lb. Because the single-turnover reactions were performed using 10-fold excess ribozyme (1000 nM) over substrate (100 nM) at concentrations that are more than an order of magnitude above the estimated Km values (40–70 nM), the substrates are assumed to have been fully bound to ribozyme. Differences in the magnesium sensitivities among RzC, RzCΔ1 and RzCΔ2 are therefore not due to relative occupancy of the enzyme, but rather to conformational differences arising from differential tertiary stabilization. It was not possible to compare affinities of the three ribozymes at 0.5 mM MgCl_2_, as only RzC was active under these conditions. The slight curvature in its Eadie-Hofstee plot could be interpreted as experimental noise, or as reflecting higher affinity at low concentrations of substrate than at high concentrations.

### Effects of sequence context on cleavage in sub-millimolar MgCl_2_

**Figure 5:**
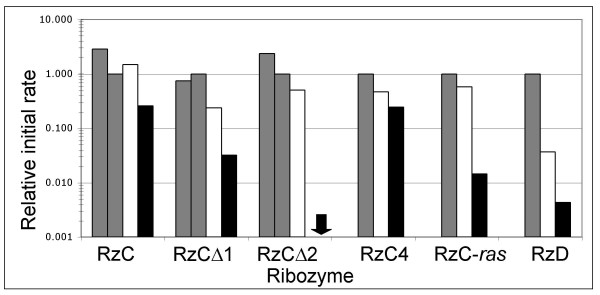
Summary of magnesium dependence of initial rates. Initial rates observed in 10 (gray bars), 5 (diagonally hatched bars), 2 (white bars) and 0.5 (black bars) mM MgCl_2 _are normalized to the rate observed in 5 mM MgCl_2_. The down arrow for RzCΔ2 indicates that no cleavage was observed under these conditions. Symbols above the bars indicate magnesium sensitivity: "++," moderate magnesium dependence indicative of tertiary stabilization; "+/-," intermediate magnesium dependence indicative of modest tertiary stabilization; "-," strong magnesium sensitivity indicative of there being no tertiary stabilization.

Two new ribozymes were generated to determine the generality of the discontinuous stem 1 hammerhead design (Figure 2). First, in construct RzC4, the individual base pairs of stems 1 and 3 in RzC were inverted to their Watson-Crick opposites except for the GUC at the cleavage site. The magnesium dependence of RzC4 was quite similar to that of RzC, declining 4-fold as MgCl_2 _was reduced from 5 mM to 0.5 mM (Figure 5, Table 2). Second, ribozyme RzC-*ras *was designed to cleave the fifteen nucleotide fragment of the human *ras *oncogene. In published cellular assays, ribozyme-mediated cleavage at this site reduced expression of a ras-luciferase fusion by 60% [18]. The 3' end of the RzC-*ras *substrate extends beyond the ribozyme, generating a branched junction within stem 1. Cleavage of the *ras *substrate by ribozyme RzC-*ras *is robust at both 2 mM and 5 mM MgCl_2_, proceeding at initial rates of 0.48 and 0.83 min^-1^, respectively, and reaching a calculated plateau of approximately 70%. At 0.5 mM MgCl_2_, the initial cleavage rate drops off sharply (40-fold reduced), although the calculated plateau is still approximately 70%, (Figure 5 and Table 2). It is possible that ribozyme RzC-*ras *initially folds into an active conformation when assayed at the highest concentrations of MgCl_2_, but that at the lowest concentrations of MgCl_2 _it initially assumes an inactive conformation. The rate-limiting step of the majority species under these conditions is then the inactive-to-active conformational conversion, which is facilitated by the presence of the TSM. In sum, several of the stabilizing features observed in RzC are also seen in other sequence contexts, including ones in which the substrate includes flanking nucleotides not paired with the ribozyme.

**Table 2:** Generalization of discontinuous hammerhead ribozyme design

	[MgCl_2_], mM	F_∞_	F_a_	k_a_	k_b_	init rate, min^-1^
RzC4 [37]	5	0.32		0.24		0.077
	2	0.27		0.13		0.036
	0.5	0.35		0.055		0.019
RzC-*ras *[37]	5	0.69		1.21		0.83
	2	0.69	0.45	1.44	0.066	0.48
	0.5	0.71		0.017		0.012
RzD [37]	5	0.52		0.125		0.065
	2	0.0135		0.0175		0.0024
	0.5	0.812		0.00032		0.00022

Generalization of discontinuous hammerhead design, following same conventions as in Table 1. Most data fit well to a single-exponential rate equation except for RzC-ras at 2 mM MgCl_2_, for which a double-exponential rate equation was used.

### Importance of stem 1b stability

The lengths of stems 1 and 2 critically determine the positioning of the interacting nucleotides at the ends of each stem. For a given stabilized hammerhead ribozyme, variants with shorter or longer stems 1 are not expected to retain tertiary stabilization – their tertiary interaction elements would be nearer to (or farther from) the core, and the nucleotides in loop 1 would be rotated by approximately 30° around the helical axis for each base pair change in helix length. The sTRSV and RzC hammerhead ribozymes have 6 total base pairs in stem 1 (three each in stems la and 1b). In contrast, the hammerhead ribozyme from peach latent mosaic viroid (PLMVd) has only five base pairs in its stem 1. In this case a three-nucleotide loop 1 (UAA) interacts with a hexaloop (UAGAGU) in loop 2 [2,4]. Ribozyme RzD was designed to determine whether hammerhead ribozymes with 5 nucleotides in stem 1 could be converted from *cis*-cleavage to *trans*-cleavage by following the design strategy used to generate ribozyme RzC. Specifically, stem 1 was divided into a three base pair stem la (to promote intermolecular substrate binding) and a two base pair stem 1b (to preserve the original PLMVd tertiary interaction). Ribozyme RzD showed markedly sharper dependence on divalent magnesium than had been observed for RzC, with initial cleavage rates declining ~300-fold as MgCl_2 _was decreased from 5 mM to 0.5 mM. The 2 bp of stem 1b thus appear to be insufficiently stable to support the discontinuous design.

### Conformational heterogeneity and generalizability of the design

It is common for hammerhead ribozymes to fold into heterogeneous populations in which a subset cleaves rapidly while the remaining RNAs convert over time into active fold which then cleave. This pattern is evident in the biphasic kinetics seen with several of the constructs described here (Figures 3 and 5; Tables 1 and 2). The tertiary stabilized, type 1 hammerhead ribozyme from the intestinal fluke parasite, *Schistosoma mansoni *(SMα1), also exhibits multiphasic kinetics indicative of conformational heterogeneity [19]. We observed similar results with the tertiary stabilized type 2 hammerhead ribozyme from the *Dolichopoda *cave cricket, and found that this heterogeneity was largely eliminated by changing a few nucleotides within stems 1 and 3 (M. Roychowdhury & D. Burke, in preparation). While conformational heterogeneity affects initial rates and the chemical interpretation of catalytic mechanism, it is of minimal relevance to modulation of gene expression by intracellularly expressed ribozymes so long as the target RNA can be cleaved fast enough to exert the desired biological effect. The discontinuous design used in ribozyme RzC yields rapid, multiple-turnover cleavage at physiological concentration of divalent magnesium, thereby demonstrating its potential utility for use inside cells.

While this manuscript was in preparation, Weinberg and Rossi described a slightly different design for hammerheads with discontinuous stems 1, in which additional base pairs are allowed to form between ribozyme and substrate by introducing a new stem at the discontinuity [20]. Although those authors only evaluated single-turnover cleavage under conditions of high divalent magnesium (10 mM), the results described here suggest that the Weinberg and Rossi design may also allow cleavage at sub-millimolar concentrations of magnesium.

## Conclusion

The "Discontinuous Stem 1" design described here takes advantage of natural stabilizing tertiary interactions in approximately native structural contexts to enable *trans*-cleavage of model substrates at physiological concentrations of MgCl_2_. The design was particularly effective for ribozymes derived from sTRSV hammerhead ribozyme, and markedly less effective for a ribozyme derived from PLMVd. Hammerhead ribozyme RzC cleaved its 13 nucleotide target substrate effectively at MgCl_2 _concentrations as low as 0.2 mM at 25°C and 0.5 mM at 37°C. Catalytic activity was reduced or eliminated at sub-millimolar MgCl_2 _concentrations for ribozymes in which tertiary interactions are diminished (RzCΔ1) or removed (RzCΔ2). Two additional ribozymes that altered the internal guide sequence, including one targeted to the human *ras *oncogene, were active in low concentrations of MgCl_2_, demonstrating that this design could be adapted for use against other targets. We envision that *trans*-cleaving ribozymes that exploit the tertiary stabilizing motifs of sTRSV or other type II or type III hammerheads could be used in gene therapies or other intracellular applications.

## Methods

### Ribozymes, RNA substrates, and oligonucleotides

RNA substrates were synthesized by Dharmacon (Lafayette, CO) and DNA oligonucleotides by Integrated DNA Technologies (Coralville, IA). Ribozymes were synthesized by transcription *in vitro *from synthetic DNA templates, then radiolabeled and purified as described [21].

### Determination of single-turnover kinetic parameters and initial rates

Kinetic analysis was carried out essentially as described [4]. Briefly, for single-turnover reactions, 10 pmol of end-labeled substrate RNA was mixed with 100 pmol ribozyme in Tris-HCl, pH 7.5 (final 50 mM in reaction), heated to 90°C for 1 min, then allowed to cool slowly to the reaction temperature. After equilibrating at either 25°C or 37°C for 5 min, one-tenth of the sample was removed as a zero time point and quenched in an equal volume of stop buffer (95% formamide, 20 mM EDTA, 0.5% bromophenol blue, 0.5% xylene cyanol). Cleavage reactions for the remainder of the sample were initiated by adding MgCl_2 _to the desired concentration. Aliquots were removed at various times and quenched in stop buffer. Cleaved and uncleaved substrate were separated on by denaturing (7 M urea) 12% polyacrylamide gel electrophoresis. Bands in the gels were quantified using the ImageQuant software from Molecular Dynamics. The data were first modeled by a single-exponential equation , where f_t _= fraction cleaved at a given time (cleaved/(cleaved + uncleaved)), f_0 _= zero point correction (essentially zero), f_∞ _= estimated plateau value at infinite time, and k_obs _= observed first-order rate constant. Those data sets that could not be adequately modeled as single-exponential processes were fit to a double-exponential process using the equation , where k_a _and k_b _are the k_obs _values for two exponentially decaying processes, and "*a*" is the fraction of the active ribozyme that partitions into the faster of the two processes. Initial cleavage rates were calculated by taking the first derivative of the single- or double-exponential equation above and solving for t = 0 to yield. For example, ignoring the f_0 _term, the initial rate for the double-exponential process is given by rate = f_∞_•a•k_a _+ f_∞_•(1-a)•k_b_. Multiple-turnover reactions were carried out in a similar manner using 1 nM ribozyme and excess substrate (10 to 200 nM).

## Abbreviations

TSM, tertiary stabilizing motif; sTRSV, satellite RNA of the Tobacco Ring-Spot Virus; PLMVd, peach latent mosaic viroid.

## Authors' contributions

STG carried out the enzyme kinetic analysis. DHB conceived of the study, participated in its design and drafted the manuscript. Both authors read and approved the final manuscript.

**Figure 4:**
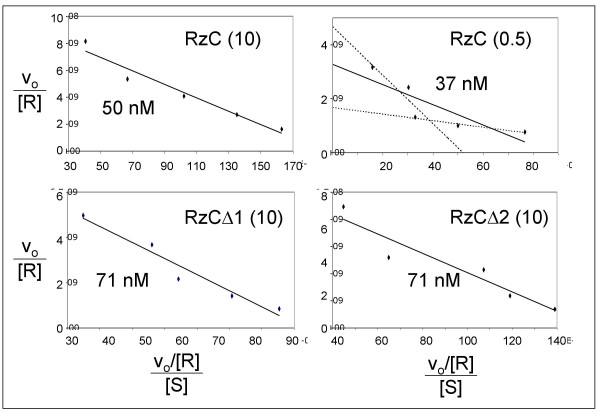
Eadie-Hofstee plots for determining substrate binding affinities. Reaction velocity (v_o_) in micromoles•min^-1 ^is normalized to total ribozyme concentration (1 nM). Indicated on each plot are the ribozyme species, the concentration of MgCl_2 _in millimolar (in parentheses) and the calculated Km value (negative of the slope). Gray trendlines for RzC at 0.5 mM MgCl_2 _subdivide the data set into regions of steep and shallow slopes with Km values of 90 nM and 12 nM.
